# Experiences and barriers to Health-Related Quality of Life following liver transplantation: a qualitative analysis of the perspectives of pediatric patients and their parents

**DOI:** 10.1186/1477-7525-8-150

**Published:** 2010-12-22

**Authors:** David B Nicholas, Anthony R Otley, Rachel Taylor, Anil Dhawan, Susan Gilmour, Vicky Lee Ng

**Affiliations:** 1 Research Institute, The Hospital for Sick Children, University of Toronto, Toronto, Ontario, Canada; 2 University of Calgary, Faculty of Social Work, Central and Northern Alberta Region, Edmonton, Alberta, Canada; 3 IWK Hospital, Dalhousie University, Halifax, Nova Scotia, Canada; 4 Department of Children's Nursing, Faculty of Health & Social Care, London South Bank University, UK; 5 King's College Hospital, London, UK; 6 Stollery Children's Hospital, University of Alberta, Edmonton, Alberta, Canada; 7 SickKids Transplant Centre, The Hospital for Sick Children, University of Toronto, Toronto, Ontario, Canada

## Abstract

This paper examines health-related quality of life (HRQOL) experiences and barriers facing young people who have received a liver transplant (LT). Semi-structured qualitative interviews were conducted with children and adolescents who have undergone LT and their parents. Findings indicate that LT fosters substantially improved child and adolescent HRQOL; however, young people also experience challenges such as difficulties with medication compliance, self-management of care routines, physical activity restrictions, and undesirable medical procedures. Implications and recommendations for clinical practice and research are discussed.

## 

Long-term survival following orthotopic liver transplantation (LT) is now the rule rather than the exception for an increasing proportion of children with chronic or acute end-stage liver disease [1]. Accordingly, assessment of outcomes after LT must consider not only the quantity of life years survived, but also the lived experience and quality of life for these children and their families. Early reports of improved quality of life after LT in children relied on the presence or absence of conventional outcomes such as growth, number of hospitalizations, and disease symptoms, without direct measurements of social, educational and emotional benefits or impairments [2]. While life saving and enhancing quality of life, LT is not curative in that a fatal disease is replaced by a chronic condition that introduces morbidities such as renal dysfunction, risk of de novo malignancies, and a life-long need for immunosuppression. These challenges are reported to impose potential burden with varying severity and impact for patients and their families [3].

Health-related quality of life (HRQOL) has emerged as an important outcome, with increasing currency in the pediatric and rehabilitation literature. The World Health Organization defines quality of life as a state of well-being rather than merely the absence of disease [4]. This definition invites a multi-dimensional approach that encompasses physical outcomes as well as life quality and perceived well-being. Until recently, most information about HRQOL among pediatric LT recipients has emerged from research in single centers, with relatively small sample sizes [5] and quantitative methodologies [6]. However, a multi-centre study recently reported on the administration of the PedsQL™, a well-validated instrument measuring HRQOL, to 363 pediatric LT patients as well as to both a normative sample and a pediatric cancer sample [7]. Pediatric LT patients reported significantly lower HRQOL relative to healthy children with absence from school and school functioning being areas of greatest discrepancy with healthy children. LT patient scores were comparable to cancer patients in social and school functioning [7]; and within other studies, pediatric LT recipients demonstrated better physical function and comparable psychosocial function relative to children with other chronic diseases [8,9]. HRQOL in children who have undergone pediatric LT appears moderately diminished compared to children without chronic illness, including lower perceptions of general health, greater emotional impact on parents, and increased intrusion on family activities [5,7-10].

In a multi-center analysis of HRQOL and family function in pediatric LT [11], parents reported significant levels of family stress, yet family function assessment scores did not yield higher levels of family dysfunction than controls [11]. It was concluded that demographic variables may have a strong impact on family function and HRQOL [11]. Taylor et al explored factors associated with HRQOL, using self-report measures to assess the influence of allograft morbidity and psychological and family variables on adolescent LT recipients' HRQOL [12]. Findings suggest that age at transplant, secondary chronic illness, symptom distress, headaches, history of emotional difficulties, self-esteem and family conflict, are factors associated with adolescent HRQOL [12].

While these studies demonstrate increased empirical understanding of HRQOL in pediatric LT, greater knowledge is needed regarding the experiences of, and perceived facilitators and barriers to, HRQOL for pediatric LT patients and their families. The objectives of this study were to explore such elements of reported HRQOL among children and adolescents with a LT.

## Methods

Qualitative interviews were conducted with young LT recipients and parents based on a *'qualitative description' *approach. In qualitative description, participants' descriptive and narrative text in interviews is upheld as "a vehicle of communication" reflecting attention to the "surface of words and events" more than consideration of theoretically-driven meanings and deeper interpretations of text [13]. This approach is relevant when relatively straight-forward description of presented phenomena is desired or likely, as in the presentation of data by young children whose developmental level may be more aligned with, or reflective of, concrete 'in the moment' description of experience.

Semi-structured interviews, each lasting approximately 0.5-1 hour, were conducted separately with children with a LT and/or a parent by a team member or research assistant experienced in qualitatively interviewing children, young people and families. Participants were interviewed in the ambulatory clinic setting or family home, at the convenience of participants; and in cases of substantial distance from the treatment hospital, participants were interviewed by telephone. Interview questions, as outlined below, addressed perceived experiences in daily life as they relate to LT and participants' perceptions of HRQOL. An interview schedule outlining open-ended questions and probes was developed following a rigorous process of literature review and pilot review of key questions from clinicians and researchers. Interviews were audio recorded and transcribed verbatim in preparation for analysis. Institutional research ethics approval was received prior to study commencement, and all participants provided written consent (and assent for those under 16 years of age) prior to study participation.

### Parent interview questions

1. Can you tell me about your child's liver transplant and what that has been like for her/him?

2. Do you remember what your child's life was like before she/he had a transplant? If yes, has anything changed since then? Has anything stayed the same?

3. What does your child like to do in her/his spare time?

4. Are these the same things he/she enjoyed before the transplant?

5. Walk me through a typical school day in your child's life? Are any parts of your child's school day affected by the fact that she/he has had a transplant? Explain.

6. Walk me through a typical weekend or non-school day in your child's life? Are any parts of your child's day affected by the fact that she/he has had a transplant? Explain.

7. Has receiving a new liver affected any other parts of your child's life?

8. Tell me about your child's daily medical management?

9. Does your child do any medical things independently? Explain.

10. Do you need to assist your child with any medical things?

11. What effects - good or bad - does your child feel from their daily medications?

12. How does your child feel about coming to the hospital for: clinic appointments? blood work? procedures such as biopsies, ultrasounds, CT scans? admissions - local and tertiary level centre (if a different facility)?

13a. Does your child have any worries that we haven't already talked about? If yes, what are they about?

13b. If you were the donor, does/did your child have any worries about your health now or at the time of transplantation?

13. Does anything bother your child about her/his health in general? If yes, what?

14. What is the worst thing for your child about having had a liver transplant?

15. What is the best thing for your child about having had a liver transplant?

16. Does your child do anything specific to stay healthy? What helps? What doesn't help?

17. Are there other important things that we haven't talked about related to your child's transplant?

### Child and adolescent interview questions

1. Do you know why you come to the hospital to see the doctor and to have blood work? (If the child knows why), do you remember your liver transplant? (If yes), can you tell me about your transplant? (Do you know why you needed a liver transplant?)

2. What's the best thing about the transplant, what's the worst thing? How do you feel?

3. (If the child remembers transplantation), can you remember what it was like before you had a transplant? (If yes), are things better or worse now than before your transplant? Explain.

4. What do you like to do for fun? (If child remembers life before the transplant), could you do the same thing before your transplant? Describe.

5. Can you tell me what you do when you go to school? Is anything hard about going school? Do you like school? Why, why not?

6. What do you do on a summer day (or a day when there is no school)?

7. Do you have friends? Explain.

8. Do you have brothers and sisters? If yes, describe.

9. Tell me about your medication? Does your mother or father help you with medications? Do you mind taking medications?

10. How do you feel about coming to the hospital to see the doctors and nurses? Do you mind having blood work? Have you had any procedures like biopsies, ultrasounds, CT scans? What was that like?

11. What kind of things do you worry about? Any problems with your transplant? Your health?

12. What kinds of things help you feel good?

13. Is there anything else you want to talk about?

#### Data Analysis

Interviews were subjected to established methods of qualitative content analysis comprising code identification, category saturation, and theme generation. A database management and computer software system for qualitative data analysis (NVivo) was utilized. A thematic analysis approach, guided by McCracken's *long interview *method, comprised a multi-step process of: (1) line-by-line review and code identification for salient constructs within individual transcripts, (2) identification of convergent and divergent codes across transcripts, (3) integration of codes across participant cohorts (children, parents), and (4) solidification of themes following the extensive review of the above three steps, demonstrating saturation of themes [14].

Trustworthiness of emerging themes, an established means for demonstrating qualitative research rigor such as credibility, auditability and fittingness [15], was implemented through established procedures. These procedures included *negative case analysis *in which the robustness of emerging themes was scrutinized by examining, within the dataset, instances of disconfirming or 'discrediting' data. *Member checking*, another marker of trustworthiness, was demonstrated by the review of themes by a subsample of patient and parent participants. These participants confirmed the viability of emergent themes relative to personal reflections on their own LT-related experiences. Finally, *peer debriefing*, comprising expert review and scrutiny of findings by key stakeholders in the field, was demonstrated. Accordingly, inter-professional clinicians with extensive experience in pediatric LT reviewed the findings and confirmed their 'fit' with clinical practice in the pediatric LT population.

The data were reviewed and coded by an analyst with extensive experience in qualitative data analysis who was further closely supervised by research team members. A sample of the data, initially reviewed by the coder and two additional 'blinded' members of the research team, achieved a high level of inter-rater reliability. Any discrepancies in codes were thoroughly examined and resolved. The data were reviewed until consensus in analysis was achieved among all coders. The primary analyst had interviewed participants at one of the data collection sites; however, was not a clinician with this population. Any potential coder bias was mitigated by an attempt to 'bracket' areas of potential coding bias, review emerging codes relative to the data, and periodic 'check ins' on the coding process by three members of the team including a qualitative methodologist.

## Results

A total of 42 pediatric patients (64% female and 36% male; average age of 12 years) and parents (84% mothers and 16% fathers) participated in qualitative interviews. All participating patients had received an isolated LT at least one year prior to study recruitment, following an earlier diagnosis most commonly of biliary atresia, acute liver failure or metabolic liver disease. They received care at a pediatric hospital in either Canada or the United Kingdom. The sample was purposively selected for diversity according to patient age, sex, cultural background, and time since transplantation. Given our data collection strategy across distinct regions (eastern Canada [Halifax], central Canada [Toronto], western Canada [Edmonton], the United Kingdom [England, Wales and Scotland] and Eire), the sample comprised diversity in region and health system, including available resources at the treatment center.

Four broad areas of thematic focus emerged: (1) LT as a facilitator of HRQOL, (2) barriers to HRQOL, (3) youth-specific perspectives, and (4) parent-specific perspectives. Each of these areas is discussed below.

### 1. LT as a Facilitator of HRQOL

LT was found to benefit children and adolescents in the areas of *health*, *physical activity*, *relationships *and *overall HRQOL *(Table 1). A majority of participants (59% of parents and children) reported no or few current problems with LT recipients' health. A high percentage of parents (70% of mothers and 72% of fathers) reported increased HRQOL for their child since transplantation, and 13% reported no change in HRQOL. Seventy one percent of participants reported that the child with a LT typically was physically active while 29% of children had limitations in sports-related activities. A total of 64% of participants reported that children with a LT had normal relationships with friends while only 9% reported difficulties for the child or adolescent in forming or maintaining friendships.

**Table 1:** Examples of participant quotes related to benefits associated with LT

*Benefit of LT*	*Quote*
Health	Eight year old boy: "(My liver transplant) doesn't make me feel anything. I'm still an ordinary kid doing what ordinary kids do. It's just that something is wrong with my liver." Parent of four year old girl: "I'm just very, very thankful that I've got a daughter who is happy and healthy, and has her whole life in front of her... So ultimately would you wish you wouldn't have had to gone through it? Yes, but having gone through it and seeing her do so well, I can't imagine our lives not having gone through that process." Parent of fifteen year old girl: "Back then it was scary (because) she was on the waiting list for three years. We didn't know when she was going to get one and it seemed she was getting sicker and sicker by the day. But now, she's been doing good for so long. It is so much easier." Interviewer: "How has her transplant affected her?" Parent: "I think it affected her in a good way because you know she can do whatever she wants to do..."

Physical Activity	Interviewer: "How has your child's transplant affected him in terms of his ability to move and play and sleep and the things that kids would normally do?" Parent of eleven year old boy: "Well it was huge. It was like his rebirth." Sixteen year old girl: "Now I'm well but before the transplant, I was out of breath a lot and I got tired really quickly. Just couldn't do things. I couldn't do sports or I couldn't get up the stairs. I had to have a rest all the time. I couldn't go to town with my friends or anything like that." Parent of seven year old boy: "I think he can do pretty much anything." Interviewer: "Is there anything else that you'd like to tell me about his life following a liver transplant?" Parent: "He's active, he does everything, like we don't limit him to anything. I mean it's up to him... He knows where his limit is. You know, he plays hockey, he enjoys hockey, he just enjoys so much, is so energetic. So it's just like he has never been sick."

Relationships	Interviewer: "Were friends supportive of you?" Seventeen year old boy: "Yes because I told them all about my transplant and they understood and they stood by me... They got to know me, so they accepted me. And all through school they were nice to me. They helped me get through a lot of things. Some of them even said, 'if anyone picks on you, talk to us about it'. They helped me get through school life." Interviewer: "How has receiving a new liver affected life?" Parent of five year old boy: "It's all improved, less restrictive with his medication and his special feedings that he used to have. He's got more time to play and it's expanded his friends, and time he can spend with them." Parent of sixteen year old girl: "As far as she's concerned, she's normal..., she's not different (than) anybody else. It's just part of her. She lets all her friends know. If she's not well and she's out with them, I know that they would ring immediately because they would look after her. But they don't treat her differently than anybody else."

Overall HRQOL	Parent of twelve year old girl: "With regards to quality of life, my daughter has been remarkably healthy and has progressed very well. In terms of her physical development, she looks normal. In terms of her stamina, I would qualify that as normal. She is a straight A student in school. She is very self-motivated. Other than the fact that she is in clinic twice a year and has medicine to take twice a day, you would never know she has challenges. She is as normal as her brother." Parent of four year old girl: "The only painful part is when they take her blood and usually, that's just a quick thing and then it is over. From my point of view, she doesn't have a lot of negative associations with the hospital or even with the whole transplant experience because luckily she hasn't really been that sick. Since the transplant, her development has caught up. She is running and jumping like a normal child her age whereas before her physical development, she was behind because of her liver disease."

### 2. Barriers to HRQOL

While recognizing substantial benefits, LT was described to also introduce challenges in daily life, with key areas of concern noted in adherence and self management of care, physical activity restriction, impact on activities of daily living, and difficult medical procedures (Table 2). Similar in proportion to the number of parent participants recognizing post LT gains in HRQOL, the majority of parents also identified worries concerning the health of their child, and many participants reported some form of medical complication in the first year post LT and/or medication side effects over time.

**Table 2:** Examples of participant quotes related to challenges associated with LT

*Challenge with LT*	*Quote*
Physical activity and restrictions in sports	1 year old boy: "My mom... doesn't want me to play after school activities like hockey (and) football... because I (might) get hit really hard in the stomach." 17 year old boy: "I can't play contact sports because there would be a good chance it could lead to bleeding." 18 year old boy: "I can't play football because that's not good... Football is too rough. It would just hurt me." Interviewer: "Does that bother you that you can't play football?" Adolescent: "Yeah... because all the other guys have good livers and I don't."

Care vigilance and restrictions in everyday activities	16 year old girl: "(There are) things I've had to watch out for after the transplant... We were planting stuff in the garden the other day and I couldn't dig in the dirt because of something in the dirt. I don't know what... the binders (instruction guides) say that I couldn't so I didn't take part in that." 8 year old girl: "I wanted to go inside my cousin's house, but her brother had the chicken pox and if I got the chicken pox, I could have died." Interviewer: "Do you think there is any part of her having a transplant that makes your life harder?" Parents of 2 year old girl: "Definitely, especially the first year post transplant. For starters, the number of medicines that she was on.... There were a lot of medicines, multiple times a day,... and then the other side of it was keeping her in isolation from anybody who had a cold..., just constantly being aware." Mother of 4 year old girl: "If I know there are people that are sick then I won't (go places)... We have had... to cancel a lot of family gatherings... You can't avoid life, but if I know someone is sick,... I can protect her as much as I can."

Difficult medical procedures	Father of 4 year old girl: "Vicariously, I can tell that she suffered a lot... We are just trying to forget, although it is very hard to forget what she has gone through... There was no spot on her body that was not punctured. There was no vein that was not touched... It was trauma followed by another trauma. It was not easy..." 15 year old girl: "A biopsy always means rejection. Well not always, but lots of times, like 90% of the time. So I have always thought of a biopsy as a bad thing." Mother of 1 year old boy: "It is upsetting... It was hard there for a while because... he'd just been extubated. They had to biopsy him and they were worried about intubation and so it is hard. He's not the easiest kid to biopsy."

Blood work: Pain and fear	11 year old girl: "I feel scared because I hate (with emphasis) needles." 17 year old girl: "This is the most useless arm for blood. You'd have to be really desperate to try. Once I had to go to the hospital for a blood test. Sometimes they'll say, 'Oh you have to have a blood test every week to sort out your medicine and everything.' Oh the joy because they're crap! I don't trust them, I really don't. And since they had to try five times, I was like screaming and crying and they wouldn't give up. And I was like, 'Give up, let me go home, I promise that I will come back tomorrow'. I was crying in the car and I then I cried when I had the blood test. Even when I had the drip in - even when they put nuclear medicine in my arm - which is cold, I never cry, but I was like, 'This is really uncomfortable, take it out'. I never cry..., but I cried and ever since then, I don't trust them... I just dread going."

In varying degrees, participants associated LT with health challenges, difficult procedures, lifestyle restrictions, and pain. They discussed the importance of pursuing health-promoting behaviors such as maintaining a healthy diet, consistency of medication administration, and exercise. Although participants recognized the importance of a healthy lifestyle, maintaining these routines was an ongoing struggle for some. Children aimed to remain healthy, yet simultaneously grappled with consequences of LT such as post-transplant complications, difficulties with medical compliance, the inconvenience of clinic visits, unwanted procedures (e.g., blood work), and worries about their health in the future.

Reflecting on barriers to HRQOL, areas of consistency and difference emerged in child and parent cohorts. Below is a discussion of these considerations, first from the vantage point of children and then from the perspective of their parents.

### 3. Child and Adolescent Perspectives

Children and adolescents with a LT reported multiple concerns in several domains of daily life, including substantial difficulty complying with health care requirements (such as clinic attendance, bloodwork and procedures), an intense fear of needles and bloodwork, complications after transplant (such as infection, pneumonia, rejection, septic shock, exhaustion, excessive bleeding, chicken pox, ear or dental problems, surgeries such as tonsils, adenoids, gall bladder, spleen removal and scar repair), and worry about the need for future medical procedures (such as a liver biopsy or CT scan).

Several children and adolescents described being restricted in sports-related activity. For example, they described wanting to play on a sports team at school, but were unable reportedly due to concern about risk of injury to the transplanted liver. Of further concern regarding health management, many children expressed concern about the difficulty to remember to take their medications, and some identified being inconvenienced and frustrated as a result of having to take medications on a daily basis.

Participants described bothersome side effects of medications including cushingoid features, dental problems, weight issues, excessive bleeding, high blood pressure, infection, low self esteem, mood swings, excess hair, kidney problems, swollen gums and osteoporosis. These challenges were reported to negatively impact on children's sense of comfort and view of self, with several children developing problematic behaviors such as unhealthy eating or excessive dieting.

Some children and adolescents described elements of shame, distaste and/or embarrassment about their post-surgical scar. For several, this concern reportedly did not ease over time, but rather become more pronounced in adolescence.

In terms of their families, several children described parental worries related to the child's health and future. While they identified these worries less frequently with the passage of time from transplantation, these findings illustrate children's awareness of and sensitivity to their parents' concerns, fears and anxieties. For some children, this vicarious knowledge of parental worry induced sadness and self-blame for imposing worry upon family members.

Beyond health functioning, children reported concern over absenteeism from school due to LT care, follow-up and medical complications. While some did not view missed school as a negative outcome in terms of compromised academic achievement, children consistently missed the social aspects of school-based relationships with peers.

### 4. Parent Perspectives

While recognizing monumental HRQOL gains due to the LT, parents identified several concerns associated with their child's ongoing LT care. Despite some agreement between mothers and fathers about specific domains of children's HRQOL, there was also diversity in expressed concerns and priority of need. In terms of agreement, the majority of mothers (87%) and all fathers reported worries about infection risk, potential for rejection, and the possible need for re-transplantation.

Parents also worried about medication side effects, child compliance with heath care regimens, and potentially needed procedures. The first year after transplantation was consistently noted by parents to be most critical and worrisome. The majority of parents stated that over time, children had less difficulty with medical compliance such as taking medication, attending clinic visits, having blood work and complying with other medical procedures. However, 36% of parents described negative aspects of medical compliance, with blood work considered to be the most difficult procedure required for medical maintenance (28%).

Parents expressed concern about potential LT impacts on their child's development and school achievement. A concern more frequently identified by mothers than fathers was children's post-operative course including transplant complications. Fathers were more frequently concerned about limitations upon children's activities of daily living such as restrictions in sports participation. Fathers' concern over sports limitations corresponded with a similar concern among several participating children and adolescents who also expressed disappointment about imposed restrictions in physical or sporting activity. While these findings raise questions of difference in priorities and perceptions between mothers and fathers, further research contrasting maternal and paternal perspectives is warranted as these differences were not the primary focus of this study. On the other hand, it is important to note that the small sample of parents participating in the study, although not representative, is reasonable given that this is an exploratory study and the qualitative research concept of saturation appeared to be achieved based on standards of qualitative rigor [15]. While comparative trends with a larger representative sample of fathers is recommended, this study provides important exploratory findings, inclusive of both mothers and fathers, that demonstrate potential similarities and differences in parental perspective.

## Discussion

This study reports the qualitative experiences of pediatric LT recipients and their parents, providing new understanding of disease-specific variables and the multi-dimensionality of elements influencing HRQOL after pediatric LT. Facilitators and barriers of HRQOL were identified in the context of a desirable state of child well-being. Finally, while children's and parent's perspectives on pediatric post LT HRQOL were generally complimentary, they were not always consistent. The voices and views of children most aptly illuminated perspectives of LT; however, the inclusion of parents' perspectives added context and richness. For instance, children's perspectives tended to relate to 'here and now' elements of life lived in the moment (e.g., pain) whereas parental perspectives often reflected nuanced and more abstract elements (e.g., perceptions of the future). Ensuring children's perspectives in understanding HRQOL emerges as important [7,10] as does the development of models for dealing with the different and perhaps shifting perspectives among children and parents.

Taillefer and colleagues identify an important challenge of bringing conceptual clarity to depictions of HRQOL, including elements, processes and outcomes [16]. Currently, there is a lack of specificity in definitions of HRQOL, with mediators and moderators remaining unclear. In this study, we elucidated themes that contribute to or inhibit overall HRQOL rather than suggesting that these themes constitute the breadth and comprehensiveness of that quality per se. Accordingly, facilitators and barriers are identified in the context of a desirable state of child well-being, defined as health, functionality, participation in activities of daily living, and other desired outcomes of transplantation.

Pediatric LT recipients are reportedly at risk for emotional, psychological, social and school challenges [7,17]. In a phenomenological study examining the lived experience of children 7 to 15 years of age, key themes related to pediatric LT comprised children feeling the same yet different relative to peers, considering extraordinary events such as hospitalization as ordinary and familiar, experiencing recurrent pain, parental concern and worry, and children globally viewing themselves as healthy yet normalcy being reported as, "a reality and illusion" [18]. Taylor et al. found that most pediatric LT recipients desired normalcy and avoided distinguishing themselves as different from others [19]. In order to achieve this normalcy a perception of wellness appears to be maintained, at times to the detriment of treatment adherence and in the hope of not being viewed as abnormal or unwell. Accordingly, the LT appears to be viewed as normal in that a routine pattern of existence is developed, yet barriers to normalcy include changed physical characteristics, medication requirements and clinic visits [18]. Tong further identifies difficulties for young patients in redefining post-transplant identity, finding normalcy, grappling with parental overprotection, navigating social adjustment, and living with restrictions in physical activities [20].

Emerging models from other areas of chronic health may offer guidance. As an example, the shifting perspectives model posits an ongoing, complex reformulation in a patient's subjective health experience [21]. Referring to this process as a continually reformulating dialectic, Paterson demonstrates that living with illness constitutes an ever-present yet ever-changing experience with illness/health elements in the foreground (e.g., feeling well) which may contradict elements that may be present in the background (e.g., critical illness requiring transplantation and vigilance in treatment adherence) [21]. This model offers relevance and flexibility by allowing individuals to make sense of their health experience; however, the contradiction of feeling well yet needing vigilant care may impose confusion, ambivalence and health risk [21,22].

Given the reported benefits of LT yet the simultaneous vulnerabilities to HRQOL, achieving best practice in assessment and intervention for pediatric LT recipients is critical. To that end, conceptual, empirical and evaluative advancement emerge as ongoing research priorities. It is expected that existing research with well-validated generic tools will be enhanced by the development of disease-specific HRQOL instruments based on direct input from pediatric LT recipients. Accordingly, finding effective ways to measure HRQOL and ultimately mitigate barriers and foster resilience, constitute important clinical and research priorities in the pursuit of HRQOL for LT recipients and their families.

## Competing interests

The authors declare that they have no competing interests.

## Authors' contributions

DBN and VLN contributed to qualitative analysis and drafted the manuscript. ARO, RT, AD and SG contributed to qualitative analysis and assisted in manuscript development. All authors approved the final manuscript.
